# Respiratory Outcomes After 6 Months of Hospital Discharge in Patients Affected by COVID-19: A Prospective Cohort

**DOI:** 10.3389/fmed.2022.795074

**Published:** 2022-03-07

**Authors:** Gabriele da Silveira Prestes, Carla Sasso Simon, Roger Walz, Cristiane Ritter, Felipe Dal-Pizzol

**Affiliations:** ^1^Laboratory of Translational Biomedicine, Graduate Program in Health Sciences, Universidade do Extremo Sul Catarinense, Criciuma, Brazil; ^2^Laboratory of Experimental Pathophysiology, Graduate Program in Health Sciences, Universidade do Extremo Sul Catarinense, Criciúma, Brazil; ^3^Department of Clinical Medicine, Center for Applied Neuroscience (CeNAp), University Hospital - UFSC, Federal University of Santa Catarina, Florianópolis, Brazil; ^4^Intensive Care Unit, Hospital São José, Criciúma, Brazil; ^5^Clinical Research Center, Hospital São José, Criciúma, Brazil

**Keywords:** pulmonary function, COVID-19, activities of daily living, mMRC scale, post COVID-19 condition, long-COVID

## Abstract

**Background:**

Considering millions of people affected by Coronavirus disease 2019 (COVID-19), long-lasting sequelae can significantly impact health worldwide. Data from prospective studies in lower-middle-income countries on persistent lung dysfunction secondary to COVID-19 are lacking. This work aims to determine risk factors and the impact of persistent lung dysfunctions in COVID-19 survivors.

**Methods:**

Observational and prospective cohort of patients admitted to a tertiary hospital from June 2020 to November 2020. Persistence of chest CT scan alterations, desaturation in the six-minute walk test (6MWT), forced expiratory volume in one second (FEV1), lung carbon monoxide diffusion (DLCO), and maximum inspiratory pressure (MIP) were measured 6 months after hospital discharge. Additionally, the Barthel index (BI) and the Modified Medical Research Council (mMRC) Dyspnea Scale were used to determine the impact of lung dysfunction in activities of daily living (ADL).

**Results:**

It was included 44 patients. Sixty percent had persistent lung CT scan abnormalities. From 18 to 43% of patients had at least one pulmonary function dysfunction, a decrease in FEV1 was the least prevalent (18%), and a reduction in DLCO and MIP was the most frequent (43%). In general, female gender, comorbidity index, and age were associated with worse lung function. Additionally, the presence of lung dysfunction could predict worse BI (r-square 0.28) and mMRC (r-square 0.32).

**Conclusion:**

Long-term lung dysfunction is relatively common in survivors from severe COVID-19 and impacts negatively on ADL and the intensity of dyspnea, similar to studies in high-income countries.

## Introduction

In addition to the characteristic symptoms of the acute infectious process of coronavirus disease 2019 (COVID-19), such as fever, cough, and chest discomfort and, in severe cases, dyspnea and bilateral pulmonary infiltration (1, 2); “post-COVID condition” reports are increasing. Still, its prevalence, risk factors, or whether one can predict the occurrence of “post-COVID condition” is not known (3, 4). The consequences of acute lung damage drove by COVID-19 could be permanent lung damage if the patient recovers (5, 6).

Until today, there is not sufficient evidence on the long-term prognosis of patients who had pneumonia due to COVID-19 (5). The inflammatory storm that characterizes severe forms of the disease suggests that serious tissue sequelae may affect various organ systems. The most common symptoms were fatigue, cognitive problems, and new-onset dyspnea. In this context, McGroder et al. (6) evaluated patients 4 months after hospitalization. Predominantly in patients who underwent mechanical ventilation, fibrotic-type patterns were observed on CT. Elderly patients are at an even higher risk, and in this population, even less severe dysfunctions can cause increased morbidity and mortality (7). Few studies suggested that this pattern persist up to 12 months after hospitalization (8–10). Like other severe conditions (11), all these sequela would impacto on the ability to perform activities of daily living (ADL) and physical capacity (12).

Thus, lung dysfunction is a critical question in “post-COVID condition”. Still, we do not have enough data on risk factors, the impact of this dysfunction on patients' daily living, mainly coming from lower-middle-income countries. In this context, we performed a prospective cohort study to identify pulmonary outcomes after 6 months of hospital discharge in patients who developed pneumonia due to COVID-19 in South Brazil. We hypothesize that 6 months after hospitalization due to COVID-19 pneumonia, lung dysfunction is frequent, and negatively affects the ADL.

## Methods

### Study Design

A prospective cohort was conducted with patients admitted to a tertiary hospital from June 2020 to November 2020. The São José hospital's Institutional Review Board approved the protocol under the number 31384620.6.1001.5364. All patients or their surrogates gave written consent before inclusion in the study.

### Setting

The study sample consisted of all consecutive patients admitted to the COVID-19 ward or intensive care unit (ICU) of a tertiary hospital from June 2020 to November 2020.

### Participants

The inclusion criteria were patients over 18 years old admitted to the hospital with confirmed COVID-19 diagnosis through reverse transcriptase reaction or rapid antigen test and requiring supplementary oxygen, non-invasive ventilation, or mechanical ventilation due to COVID-19 pneumonia. Exclusion criteria were patients with severe chronic diseases (chronic kidney disease under dialysis, cirrhosis child C, severe COPD, severe heart failure) or diseases capable of altering inflammatory response (such as chronic use of immunosuppressants, cancer patients without disease control, and HIV without disease control), and patients in palliative care or with life expectancy <24 h.

### Procedures

Investigators daily screened all patients admitted to the hospital, and those who met the inclusion criteria were considered eligible. The patient was invited to participate in the study from hospital admission for a maximum of 120 h. All necessary information was prospectively collected directly from the patient's electronic medical record. Prehospital comorbidities were aggregated through the validated Charlson comorbidity index. The severity of critical illness at ICU admission was collected with the Simplified Acute Physiology Score (SAPS) 3. The Sequential Organ Failure Assessment (SOFA) score was used to assess organ dysfunction. A chest CT scan was performed on admission and was analyzed to determine the extent of pulmonary involvement. Approximately, 6 months after hospital discharge, patients attended the hospital's outpatient clinic. They were evaluated with chest CT scan, lung carbon monoxide diffusion (DLCO), and lung plethysmography, a six-minute walk test (6MWT), ADL, and dyspnea intensity, performed as described below. The outcome evaluation was blinded for the hospitalization variables.

### Chest Computed Tomography

The extension of acute-phase ground-glass opacity was graded as <25%, 25–50%, and >50% at hospital admission, modified from Guan et al. (13). At 6-months after hospital discharge, the persistence of ground-glass and the occurrence of lung fibrosis were evaluated.

### Lung Plethysmography With Carbon Monoxide Diffusion

Pulmonary function tests were done according to the American Thoracic Society (ATS)–European Respiratory Society guidelines. A variable pressure VIASYS Respiratory Care plethysmography (Vyaire, Mettawa, IL, USA) was used to determine the following parameters: FEV1, DLCO, and maximum inspiratory pressure (MIP). For FEV1, flow-volume curves were obtained and the greatest percentage of the three maneuver was used for analysis. For MIP, each participant was asked to perform five manoeuvers, with a goal of matching the highest two within 10 cm H_2_O, and the largest MIP from each participant's test was used for analysis. These variables were expressed as percentages of predicted normal values. Normal values were considered those ≥80% of predicted values (6).

### Six-Minute Walk Test

Each patient walked on the flat ground as fast as possible without oxygen inhalation and completed the 6MWT independently. From the 6MWT, significant oxygen desaturation was the parameter used to qualify the patient's performance, defined as a decrease of at least 4% from baseline SpO_2_ (14).

### The Barthel Index

The BI is a ten-item ordinal scale used to measure performance ADL (15). BI scored according to the level of physical assistance required to perform the daily task.

### The Modified Medical Research Council Dyspnea Scale

The Modified Medical Research Council (mMRC) Dyspnea Scale is a self-rating tool to measure the degree of disability that breathlessness poses on day-to-day activities on a scale from 0 to 4 (16).

### Outcomes

Outcomes were different aspects of lung function 6-months after hospital discharge: persistence of CT scan alterations, desaturation in the 6MWT, FEV1, DLCO, and MIP. Additionally, the BI and the mMRC were used to determine the impact of lung dysfunction in ADL.

### Statistical Analysis

The collected data were analyzed in the IBM SPSS Statistics version 22.0 software (IBM Corp., Armonk, N.Y., USA). Quantitative variables were expressed as mean and standard deviation, and were compared using the Student's *t*-test. Nominal variables were expressed as frequency and percentage, and were compered using the Pearson's chi-square. The logistic binary regression was used to access the independent risk factors for the presence of lung dysfunction. Lung function parameters were dichotomized as described above, and the predictive variables were entered in the model as continuous or categorical depending on their characteristics. The model included only variables with *p* < 0.20 or *p* < 0.05 in the univariate analysis depending on the number of events observed in each outcome to not overfit the model. If variables that reached the threshold had collinearity, the variable with a lower *p*-value in the univariate analysis entered the final model. Results from univariate analysis were presented as *p*-value and logistic binary regression as relative risk and 95% CI. Linear regression was performed to determine the impact of lung dysfunction on ADL, and R squared was calculated to express the percentage of the variance in the ADL that the lung dysfunction variables explained. In all analyses, a *p*-value <0.05 was adopted as the level for statistical significance.

## Results

Based on the predefined inclusion and exclusion criteria, the final sample resulted in 167 patients. From these, 56 patients died during hospitalization or follow-up. Due to the importance of a timely description of lung abnormalities, we evaluated only the first 44 consecutively included patients 6 months after hospital discharge. There were no missing cases. All patients will have a 1-year evaluation, as defined in the original protocol.

Table 1 described demographic information. Approximately, 70% of the patients were male, and the mean age was 54 ± 11 years. The mean body mass index was 30 ± 5, and the median Charlson comorbidity index was 2 (1, 2). The mean length of ICU stay was 12 ± 9.1 days and the mean length of hospital stay was 16 ± 10.3 days. The need for mechanical ventilation was 23% of the sample. The mean SAPS III score was 48 ± 12, and the mean respiratory SOFA was 2.8 ± 1 at admission (D1) and 2.0 ± 1.4 72 h after (D3). These variables were not statistically different compared to the remaining 67 patients not included in this preliminary analysis.

**Table 1:** General patients' characteristics.

**Variables**	***N*(%)**	**Mean (SD)/median (25–75)**
Age, years		54 (11)
**Gender**		
Male	31 (70)	
Female	13 (30)	
BMI		30 (5.0)
**Extent of lung involvement at admission**		
25–50%	16 (41)	
>50%	23 (59)	
Charlson comorbidity index		2 (1, 2)
Corticosteroids, yes	40 (91)	
SAPS III		48 (12.2)
**Respiratory SOFA**		
D1		2.8 (1.0)
D3		2.0 (1.4)
**SOFA**		
D1		3.7 (1.9)
D3		2.9 (2.6)
**C-reactive protein, mg/L**		
D1		118 (84)
D3		108 (96)
**ICU admission**		
Yes	31 (70)	
No	13 (30)	
ICU length of stay, days		12 (9.1)
**Invasive mechanical ventilation**		
Yes	10 (23)	
No	34 (77)	
Days on mechanical ventilation		13 (7.7)
Length of hospital stay, days		16(10.3)

*SD, standard deviation; BMI, Body mass index; SAPS III, Simplified Acute Physiology Score 3; SOFA, sequential organ failure assessment score; ICU, intensive care unit; N, number of participants*.

Lung CT scan abnormalities were ground-glass (15 from 24) and fibrosis (9 from 24). Thus, 24 (60%) of the patients had persistent lesions on CT scan. Table 2 presented the relation of acute-phase variables and the persistence of lung abnormalities on the CT scan 6-months after hospital discharge. In the univariate analysis, no single variable was associated with CT-scan lesions. However, c-reactive protein (CRP) at D1 and D3 and respiratory SOFA at D3 reached the threshold and were included in the regression analysis. Only CRP levels were marginally, but not significantly, related to the persistence of CT-scan lesions (Table 2).

**Table 2:** Variables associated with the persistence of lesions on lung CT scan 6-months after hospital discharge.

**Variables**	**Present** **(*n* = 24)**	**Absence** **(*n* = 20)**	***p*-value^a^**	**RR** **(CI 95%)^b^**
Age, years, mean (SD)	54 (11)	54 (11)	0.99	NA
**Gender**				
Male, *n* (%)	16 (66)	15 (75)	0.55	NA
BMI, mean (SD)	31 (4.8)	30 (5.4)	0.64	NA
**Extent of lung involvement at admission^c^** ***n*** **(%)**				
25–50%	8 (38)	7 (41)	0.85	NA
>50%	13 (62)	10 (59)		
Charlson Comorbidity Index, median (25–75)	2 (1, 2)	1 (1, 2)	0.34	NA
SAPS III, mean (SD)	47 (14.4)	48 (10.6)	0.91	NA
**Respiratory SOFA**				
D1, mean (SD)	2.9 (1.1)	2.7 (0.9)	0.56	NA
D3, mean (SD)	2.4 (1.3)	1.8 (1.5)	0.16^*^	1.04 (0.7–1.5)
**SOFA**				
D1, mean (SD)	3.6 (1.7)	3.9 (2.2)	0.59	NA
D3, mean (SD)	3.0 (2.5)	2.7 (2.8)	0.67	NA
**C-reactive protein, mg/L**				
D1, mean (SD)	99 (72)	146 (96)	0.13	NA
D3, mean (SD)	79 (54)	164 (133)	0.08^*^	0.98 (0.98–1.0)
ICU yes, *n* (%)	31 (55)	14 (45)	0.95	NA
ICU length of stay, days mean (SD)	12 (9.9)	11 (8.3)	0.65	NA
Mechanical ventilation, yes, *n* (%)	4 (40)	6 (60)	0.29	NA
Days on Mechanical Ventilation, mean (SD)	14 (8.2)	12 (7.9)	0.63	NA
Length of hospital stay, days, mean (SD)	17 (11.4)	15 (8.9)	0.61	NA

*SD, standard deviation; RR, Relative Risk; CI, confidence interval; NA, not applied; BMI, Body mass index; SAPS III, Simplified Acute Physiology Score 3; SOFA, sequential organ failure assessment score; ICU, intensive care unit; N, number of participants*.

* *p < 0.20 variables included in the binary logistic regression*.

a *p-value from the univariate analysis*.

b *RR form the regression analysis*.

c *for six patients, CT scan, was not performed at hospital admission*.

In the 6MWT (Table 3), 15 (35%) patients had a significant desaturation. In the univariate analysis, desaturation was associated with female gender, Charlson comorbidity index, and respiratory SOFA D3. Only the female gender was independently associated with desaturation in 6MWT. Of these 15 patients, 10 (66%) had persistence of CT scan alterations, being 6 (60%) ground-glass and 4 (40%) fibrotic lesions.

**Table 3:** Variables associated with desaturation in the six-minute walk test (6MWT) 6-months after hospital discharge.

**Variables**	**Present** **(*n* = 15)**	**Absence (*n* = 29)**	***p*-value^a^**	**RR** **(IC 95%)^b^**
Age, years, mean (SD)	56 (10)	52.7 (11)	0.24	NA
**Gender**				
Male, *n* (%)	6 (40)	25 (86)	0.001^*^	0.13 (0.03–0.6)
BMI, mean (SD)	32 (6.8)	30 (3.8)	0.52	NA
**Extent of lung involvement at admission^c^** ***n*** **(%)**				
25–50%	4 (27)	12 (50)	0.08	NA
>50%	11 (73)	12 (50)		
Charlson Comorbidity Index, median (25–75)	1 (1, 2)	2 (1–3)	0.044^*^	1.5 (0.8–2.9)
SAPS III, mean (SD)	53 (15.0)	46 (10.1)	0.15	NA
**Respiratory SOFA**				
D1, mean (SD)	2.9 (1.2)	2.7 (0.9)	0.50	NA
D3, mean (SD)	2.6 (1.1)	1.8 (1.5)	0.045^*^	1.06 (0.8–1.4)
**SOFA**				
D1, mean (SD)	3.6 (1.7)	3.6 (1.7)	0.73	NA
D3, mean (SD)	3.6 (2.8)	2.4 (2.4)	0.15	NA
**C-reactive protein, mg/L**				
D1, mean (SD)	88 (45)	136 (96)	0.07	NA
D3, mean (SD)	91 (89)	122 (102)	0.40	NA
ICU, yes, *n* (%)	11 (73)	20 (69)	0.76	NA
ICU length of stay, days, mean (SD)	14 (8.0)	10 (9.6)	0.279	NA
Mechanical ventilation, yes, *n* (%)	4 (27)	6 (21)	0.65	NA
Days on mechanical ventilation, mean (SD)	15 (5.3)	11 (9.2)	0.750	NA
Length of hospital stay, days, mean (SD)	20 (10.3)	14 (9.9)	0.485	NA

*SD, standard deviation; RR- Relative Risk; CI, confidence interval; NA, not applied; BMI, Body mass index; SAPS III, Simplified Acute Physiology Score 3; SOFA, sequential organ failure assessment score; ICU, intensive care unit; N, number of participants*.

* *p < 0.20 and included in the binary logistic regression*.

a *p-value from the univariate analysis*.

b *RR form the regression analysis*.

c *For five patients, CT scan was not performed at hospital admission*.

Only 8 (18%) of the patients had a significant (<80% of predicted value) decrease in FEV1. When analyzing the FEV1 (Table 4), age was significantly associated with an abnormal FEV1. Interestingly, all patients that had reduced FEV1 had more than 50% of ground-glass at hospital admission. However, due to the low number of events, every attempt to perform a binary regression resulted in an overfitted model. Of these eight patients, 2 (25%) had persistence of CT scan alterations, being 1 (50%) ground-glass and 1 (50%) fibrotic lesions.

**Table 4:** Variables associated with a decreased forced expiratory volume in one second 6-months after hospital discharge.

**Variables**	**Present** **(*n* = 8)**	**Absence (*n* = 36)**	***p*-value^a^**
Age, years, mean (SD)	60 (5)	53 (11)	0.015^*^
**Gender**			
Male, *n* (%)	6 (75)	25 (10)	0.75
BMI, mean (SD)	32 (5.4)	30 (5.0)	0.35
**Extent of lung involvement at admission^b^** ***n*** **(%)**			
25–50%	0 (0.0)	15 (48)	0.029^*^
>50%	7 (100)	16 (52)	
Charlson Comorbidity Index, median (25–75)	1 (1, 2)	2 (2)	0.11
SAPS III, mean (SD)	50 (15.2)	48 (12.0)	0.71
**Respiratory SOFA**			
D1, mean (SD)	2.9 (1.2)	2.8 (0.9)	0.80
D3, mean (SD)	2.0 (1.4)	2.1 (1.4)	0.87
**SOFA**			
D1, mean (SD)	3.9 (2.8)	3.7 (1.7)	0.81
D3, mean (SD)	2.6 (2.1)	2.9 (2.8)	0.76
**C-reactive protein, mg/L**			
D1, mean (SD)	108 (103)	122 (80)	0.70
D3, mean (SD)	129 (172)	103 (70)	0.72
ICU yes, *n* (%)	5 (62)	26 (72)	0.59
ICU length of stay, days, mean (SD)	14 (11.6)	11 (8.7)	0.51
Mechanical ventilation, yes, *n* (%)	2 (25)	8 (22)	0.86
Days on mechanical ventilation, mean (SD)	18 (7.8)	11 (7.4)	0.25
Length of hospital stay, days, mean (SD)	19 (11.3)	15 (10.0)	0.35

*SD, standard deviation; BMI, Body mass index; FEV1, forced expiratory volume in one second; SAPS III, Simplified Acute Physiology Score 3; SOFA, sequential organ failure assessment score; ICU, intensive care unit; N, number of participants*.

a *p-value from the univariate analysis*.

b *For six patients CT scan was not performed at hospital admission*.

* *Due to the low number of events, it was not possible to perform binary regression analysis*.

When analyzing DLCO (Table 5), 19 (43%) of patients presented a significant decrease. In the univariate analysis, age, gender, and Charlson comorbidity index were significantly associated with a reduction in DLCO, but only gender was independently associated with a decrease in diffusion. Of these 19 patients, 11 (58%) had persistence of CT scan alterations, being 6 (54%) ground-glass and 5 (36%) fibrotic lesions.

**Table 5:** Variables associated with a decreased carbon monoxide diffusion 6-months after hospital discharge.

**Variables**	**Present** **(*n* = 19)**	**Absence** **(*n* = 25)**	***p*-value^a^**	**RR (IC 95%)^b^**
Age, years, mean (SD)	59 (9)	50 (11)	0.008^*^	1.03 (0.9–1.2)
**Gender**				
Male. *n* (%)	9 (47)	22 (88)	0.003^*^	0.7 (0.006–0.7)
BMI, mean (SD)	30 (5.6)	31 (4.7)	0.73	NA
**Extent of lung involvement at admission^c^** ***n*** **(%)**				
25–50%	5 (36)	10 (42)	0.72	NA
>50%	9 (64)	14 (58)		
Charlson Comorbidity Index, median (25–75)	1 (1, 2)	2 (1–3)	0.02^*^	1.9 (0.65–5.6)
SAPS III, mean (SD)	48 (10.6)	48 (14)	0.88	NA
**Respiratory SOFA**				
D1, mean (SD)	2.68 (1.00)	2.88 (0.97)	0.51	NA
D3, mean (SD)	2.32 (1.25)	1.87 (1.51)	0.30	NA
**SOFA**				
D1, mean (SD)	3.3 (2.2)	4.0 (1.6)	0.21	NA
D3, mean (SD)	3.0 (2.0)	2.8 (3.1)	0.88	NA
**C-reactive protein, mg/L**				
D1, mean (SD)	96 (63)	137 (96)	0.16	NA
D3, mean (SD)	83 (51)	132 (121)	0.16^*^	0.99 (0.97–1.005)
ICU, yes, *n* (%)	14 (74)	17 (68)	0.68	NA
ICU length of stay, days, mean (SD)	12 (6.8)	11 (10.8)	0.68	NA
Mechanical ventilation, yes, *n* (%)	3 (16)	7 (28)	0.34	NA
Days on mechanical ventilation, mean (SD)	13 (5.5)	12 (8.8)	0.87	NA
Length of hospital stay, days, mean (SD)	17 (8.6)	15 (11.4)	0.48	NA

*SD, standard deviation; RR- Relative Risk; CI, confidence interval; NA, not applied; BMI, Body mass index; SAPS III, Simplified Acute Physiology Score 3; SOFA, sequential organ failure assessment score; ICU, intensive care unit; N, number of participants*.

a *p-value from the univariate analysis*.

b *RR form the regression analysis*.

c *For six patients, CT scan was not performed at hospital admission*.

* *p < 0.20 variables included in the binary logistic regression*.

Maximum inspiratory pressure assessed respiratory muscle strength and was decreased in 19 (43%) patients (Table 6). Age, gender, and Charlson comorbidity index were associated with reduced MIP in the univariate analysis, but no one variable was independently associated with this outcome. It was observed a marginal, non-significant association with gender and Charlson comorbidity index. Of these 19 patients, 11 (58%) had persistence of CT scan alterations, being 7 (64%) ground-glass, and 4 (36%) fibrotic lesions.

**Table 6:** Variables associated with a decreased maximum inspiratory pressure 6-months after hospital discharge.

**Variables**	**Present** **(*n* = 19)**	**Absence** **(*n* = 25)**	***p*-value^a^**	**RR** **(IC 95%)^b^**
Age, years, mean (SD)	57 (9.0)	51 (11.6)	0.05^*^	0.96 (0.86–1.08)
**Gender**				
Male. *n* (%)	10 (53)	20 (80)	0.03^*^	0.25 (0.05–1.16)
BMI, mean (SD)	30 (6.0)	31 (4.4)	0.71	NA
**Extent of lung involvement at admission^c^** ***n*** **(%)**				
25–50%	7 (40)	9 (41)	0.96	NA
>50%	9 (60)	13 (59)		
Charlson Comorbidity Index, media (25–75)	1 (1, 2)	2 (1–3)	0.008^*^	2.6 (0.8–8.0)
SAPS III, mean (SD)	45 (10.0)	50 (13.6)	0.36	NA
**Respiratory SOFA**				
D1, mean (SD)	2.8 (0.8)	2.7 (1.1)	0.65	NA
D3, mean (SD)	2.3 (1.2)	1.9 (1.6)	0.36	NA
**SOFA**				
D1, mean (SD)	3.9 (1.6)	3.6 (2.2)	0.71	NA
D3, mean (SD)	3.0 (2.1)	2.9 (3.1)	0.78	NA
**C-reactive protein, mg/L**				
D1, mean (SD)	138 (70)	100 (97)	0.22	NA
D3, mean (SD)	97 (53)	125 (128)	0.45	NA
ICU, yes, *n* (%)	14 (74)	16 (64)	0.62	NA
ICU length of stay, days, mean (SD)	10 (7.4)	13 (10.7)	0.42	NA
Mechanical ventilation, yes, *n* (%)	5 (26)	5 (20)	0.67	NA
Days on mechanical ventilation, mean (SD)	9 (8.1)	16 (6.1)	0.21	NA
Length of hospital stay, days, mean (SD)	16 (8.3)	16 (11.6)	0.92	NA

*SD, standard deviation; RR- Relative Risk; CI, confidence interval; NA, not applied; BMI, Body mass index; SAPS III, Simplified Acute Physiology Score 3; SOFA, sequential organ failure assessment score; ICU, intensive care unit; N, number of participants*.

a *p-value from the univariate analysis*.

b *RR form the regression analysis*.

c *For six patients CT scan was not performed at hospital admission*.

* *p < 0.20 variables included in the binary logistic regression*.

The impact of these dysfunctions on the BI was measured. Both the presence of desaturation on 6MWT (mean BI 91 ± 25 vs. 74 ± 27) and reduced VEF1 (mean BI 89 ± 24 vs. 68 ± 32) were significantly associated with lower BI scores (*p* < 0.05). DLCO was marginally but not significantly associated with lower BI scores (mean BI 92 ± 23 vs. 76 ± 30, *p* = 0.06). There was no association between MIP and the extension of CT lesions and BI scores. When desaturation on 6MWT, FEV1, and DLCO entered in a linear regression only FEV1 (*p* = 0.019) and marginally DLCO (*p* = 0.06) were associated with lower BI (R square for the model 0.28).

Additionally, both the presence of desaturation on 6MWT (mean mMRC 0.8 ± 0.8 vs. 1.7 ± 1.4) and reduced DLCO (mean mMRC 0.8 ± 1.2 vs. 1.6 ± 1.0) were significantly associated with higher mMRC scores (*p* < 0.05). FEV1 and the extension of CT lesions were marginally, but not significantly associated with higher mMRC scores (mean mMRC 1.0 ± 1.0 vs. 1.6 ± 1.7, *p* = 0.17 for FEV1; 0.9 ± 1.1 vs. 1.3 ± 1.2, *p* = 0.17 for CT lesions). There was no association between MIP and mMRC scores. When desaturation on 6MWT, FEV1, DLCO, and CT lesions entered in a linear regression only FEV1 (*p* = 0.017) and marginally DLCO (*p* = 0.12) were associated with higher mMRC scores (R square for the model 0.32).

## Discussion

Confirming our hypothesis, respiratory dysfunction 6 months after hospital discharge from COVID-19 was common and negatively impacted the ADL.

Lung CT scan were abnormal in more than half of the patients, and this is consistent with other studies (6, 8). Interestingly, even variables such as mechanical ventilation and respiratory SOFA were not a risk factor associated with abnormal CT scan at 6 months. Unlike González et al. (17) demonstrated that the persistence of lung abnormalities on CT scan was associated with the length of invasive mechanical ventilation. Dyspnea was attributed to abnormalities on lung CT scan (17), and this is similar to our results; half of the patients who presented lung dysfunction measured by the 6MWT, DLCO, and MIP had persistent CT scan abnormalities.

Results for abnormal lung function assessed by the 6MWT, FEV1, DLCO, and MIP showed that a considerable proportion (18–43%) had at least one alteration. DLCO and MIP were the most prevalent dysfunction observed; thus, intrinsic lung function and respiratory muscle strength were affected after COVID-19. A systematic review evaluated pulmonary function after COVID-19 (18). It included three hundred eighty patients in the data synthesis. In the sensitivity analysis, the study found a prevalence of 0.39 (*CI* 0.24–0.56, *p* < 0.01, *I*^2^ = 86%), 0.15 (*CI* 0.09–0.22, *p* = 0.03, *I*^2^ = 59%), and 0.07 (CI 0.04–0.11, *p* = 0.31, *I*^2^ = 16%) for altered DLCO, restrictive pattern and obstructive pattern, respectively, consistent with our findings. As previously demonstrated (9, 10), an increased risk for lung dysfunction was observed in women. In our sample, female sex was an independent risk factor for decreasing 6MWT and DLCO. Additionally, this was also true to MIP in the univariate analysis. Interestingly, confirming previous reports (19), female sex was not significantly associated with persistent CT-scan abnormalities; neither is persistent CT-scan abnormalities universally present in lung dysfunction patients, suggesting that distinct mechanisms could be related to these outcomes. Further biomarkers studies could help to understand the underlying mechanisms that drive these alterations.

Other risk factors associated with lung dysfunction at 6-months that were statistically significant in the univariate analysis were Charlson comorbidity index and age. This is an exciting finding suggesting that for “post-COVID condition” premorbid characteristics are more important as a risk factor when compared to variables associated with the COVID-19 acute phase. This is different when compared to Huang et al. findings (10). They found that impaired DLCO was most prevalent in patients with more severe illnesses. The population in Huang's study included less severe COVID-19 patients when compared to our study, and this could partially explain these differences.

These dysfunctions seem to be of clinical relevance since they are associated with a decreased BI and a higher degree of dyspnea assessed by the mMRC. It is important to note that the linear regression model explained 28 and 32% of the alterations in the ADL and mMRC, respectively. Thus, besides lung dysfunction, others factors associated with “post-COVID condition” could impact ADL impairment. Huang et al. (10) found that fatigue and muscle weakness were common 6-months after the onset of COVID-19 symptoms, and such symptoms could impact ADL and mMRC. Further studies should be performed to determine clinical factors associated with long-term limitations in ADL and dyspnea intensity.

This study has several limitations. First, due to the urgent need for data, we anticipated the evaluation for these 44 patients to 6-months after hospital discharge. The small sample size may result in false-negative results. However, the significant associations that were found, together with the prospective design with a blind analysis, strengthen the results' credibility. Initially, these patients would be followed up only by phone interview until 12-months after hospital discharge. Follow-up keeps going, and all patients will have a complete evaluation at 12-months after hospital discharge. Second, due to epidemiologic characteristics of the pandemia, it was not possible to include SARS patients of other etiologies such as influenza.

## Conclusion

Long-term lung dysfunction is relatively common in survivors from severe COVID-19 and impacts negatively on ADL and the intensity of dyspnea, similar to studies in high-income countries. These results highlight the need to develop strategies to prevent and treat the burden of disease associated with COVID-19.

## Data Availability Statement

The raw data supporting the conclusions of this article will be made available by the authors, without undue reservation.

## Ethics Statement

The São José Hospital's Institutional Review Board approved the protocol under the number 31384620.6.1001.5364. The patients/participants provided their written informed consent to participate in this study.

## Author Contributions

GP had full access to all of the data in the study and contributed to the study design, data collection and interpretation, and writing of manuscript. CS contributed to data collection and literature search. RW and CR contributed to the study design and to writing of the manuscript. FD-P contributed to the study design, data interpretation, and writing of manuscript. All authors contributed to the article and approved the submitted version.

## Funding

This work was supported by MCTIC/CNPq/FNDCT/MS/SCTIE/DECIT, 07/2020, grant number 401263/2020-7 and BRF S.A. Hub unrestricted donation.

## Conflict of Interest

The authors declare that the research was conducted in the absence of any commercial or financial relationships that could be construed as a potential conflict of interest.

## Publisher's Note

All claims expressed in this article are solely those of the authors and do not necessarily represent those of their affiliated organizations, or those of the publisher, the editors and the reviewers. Any product that may be evaluated in this article, or claim that may be made by its manufacturer, is not guaranteed or endorsed by the publisher.

## Abbreviations

6MWT: 6-minute walk test
ADL: Activities of daily living
BI: Barthel index
CT: Computed tomography
CRP: c-reactive protein
FEV1: Forced expiratory volume in one second
ICU: Intensive care unit
DLCO: Lung carbon monoxide diffusion
MIP: Maximum inspiratory pressure
mMRC: Modified medical research council
SOFA: Sequential Organ Failure Assessment
SAPS: Simplified Acute Physiology Score.
